# Student satisfaction and loyalty in Denmark: Application of EPSI methodology

**DOI:** 10.1371/journal.pone.0189576

**Published:** 2017-12-14

**Authors:** Tina Shahsavar, Frantisek Sudzina

**Affiliations:** Department of Business and Management, Aalborg University, Aalborg, Denmark; University of Westminster, UNITED KINGDOM

## Abstract

Monitoring and managing customers’ satisfaction are key features to benefit from today’s competitive environment. In higher education context, only a few studies are available on satisfaction and loyalty of the main customers who are the students, which signifies the need to investigate the field more thoroughly. The aim of this research is to measure the strength of determinants of students’ satisfaction and the importance of antecedents in students’ satisfaction and loyalty in Denmark. Our research model is the modification of European Performance Satisfaction Index (EPSI), which takes the university’s image direct effects on students’ expectations into account from students’ perspective. The structural equation model of student satisfaction and loyalty has been evaluated using partial least square path modelling. Our findings confirm that the EPSI framework is applicable on student satisfaction and loyalty among Danish universities. We show that all the relationships among variables of the research model are significant except the relationship between quality of software and students’ loyalty. Results further verify the significance of antecedents in students’ satisfaction and loyalty at Danish universities; the university image and student satisfaction are the antecedents of student loyalty with a significant direct effect, while perceived value, quality of hardware, quality of software, expectations, and university image are antecedents of student satisfaction. Eventually, our findings may be of an inspiration to maintain and improve students’ experiences during their study at the university. Dedicating resources to identified important factors from students’ perception enable universities to attract more students, make them highly satisfied and loyal.

## Introduction

There is a wide range of products and services that satisfy a certain need. Customers choose and buy based on their expectations of the value of products and services that are presented by different markets and the level of satisfaction they get from it. Positive word of mouth and repurchase are acts of satisfied customers with good experience. Marketers should consider the right level of expectation; too low level of expectation fails to attract enough customers and if they set it too high, customers will be disappointed. The key elements to develop and manage customer relationships are customer value and customer satisfaction. In other words, “a good customer relationship management creates customer satisfaction”. (page 19) Consequently, satisfied customers positively talk about the company and its services or products and stay loyal. On the contrary, dissatisfied customers criticize the product or service to others and they usually turn to competitors. There is a considerable difference in the loyalty of customers with different level of satisfaction. Study shows that even an insignificant drop in complete satisfaction cause a huge drop in loyalty [1]. Although Oliver [2, 3] defines loyalty in general as “deeply held commitment to repatronize a preferred product or service”, in case of higher education applies Østergaard and Kristensen's [4] definition of student loyalty as "willingness to recommend the institution and the programmes to others, considerations whether the student would have chosen the same institution and programme today, and willingness to continue education or participate in conferences at the institution in the future" or a similar definition by Temizer and Turkyilmaz [5] "as the tendency of a student to choose same provider (i.e. HEIs) or service over another for a particular need."

The increasingly competitive and dynamic educational environments brings up numerous challenges, such as possibility of declining enrolments and general public demands against the competitors. Universities are facing changes in higher education market conditions and they are responsible for their performance, which raises a need to constantly adapt their performance to standards and international demands like “The Bologna Process”. The environment that universities operate under is getting more similar to the private companies market conditions, which makes universities to become more aware of the importance of student satisfaction [4]. Student satisfaction can be understood as a short-term attitude resulting from the evaluation of the student educational experience. Elliot and Healy (2001) and Vázquez et al. (2015) have studied students as customers or clients. In their studies, students’ satisfaction measurement in higher education follows the same methodology used in general customer satisfaction measurement [6, 7]. Studies that show student satisfaction has a positive impact on student motivation, student retention, recruiting efforts and fundraising emphasize the importance of student’s satisfaction [8]. As a result, universities have exhibited their commitment to student satisfaction through mission statements, goals/objectives, marketing strategies, and promotional themes. A drop in student retention without a compensating enrolment increase affects various customers of higher education universities or institutions such as students, staff, faculty, alumni, donors, and others, which drives the need for managing loyalty and retention process from the beginning of students’ entrance till their graduation [9]. It is proven that attracting a new customer costs more than retaining one [10]. Loyalty as a crucial factor for the success of every business including colleges and universities emphasizes the need for extending the duration of the relationship with the end users, which in our case are students. The efficiencies that result from increased student loyalty cause not only increased lifetime tuition revenues from students but also provide some synergy for enrolment through low-cost word of mouth recommendation activities [11].

Although the literature on the topic of customer satisfaction and loyalty is very rich, there are only a few studies on satisfaction and loyalty from students’ perspective in higher education. In the context of higher education, the focus has often been on investigating the link between teaching quality/learning outcomes and student satisfaction/loyalty or other relationships separately. Moreover, when customers are more involved in the production process, the co-production of services will be the greatest concern in any organisation [12]. The importance of customer involvement in the higher education context becomes evident considering students as the main determinants of the service’s success, whose participation is extremely essential. Thus, monitoring and managing the quality of service constantly from students’ perspective is required.

All in all, achieving quality is an imperative goal of higher education universities, colleges or institutions. Therefore, investigating strengths and weaknesses of different aspects of service quality and design the best possible service is required, which leads to better allocation of resources and provides an improved service to students as an antecedent to student satisfaction [13]. Most of the higher education institutions are regulated by the Ministry of Higher Education and Science in Denmark. The higher education system is mainly financed by the State and the tuition is free of charge for the EEA (The European Economic Area) students. The EEA includes EU countries and also Iceland, Liechtenstein and Norway. They endeavour to create a system of high quality education, which offers the best possible opportunities and challenges for all students; a system which supports highly skilled and employable graduates for the benefit of both the individual and for the society, which will contribute to growth, productivity and prosperity in Denmark [14]. Moreover, Danish universities get paid for each student only after the student successfully finalized studies. Therefore, loyalty in the form of retention is important for universities in order to secure as high funding from the State as possible.

In this paper, we aimed to construct a comprehensive model of student satisfaction and loyalty through structural equation modelling. The objective of our research is to test the modified European Customer Satisfaction Index model, which aims to describe the extent to which students intend to remain at university, which is influenced by their satisfaction of university, university’s image, quality of software and hardware, perceived value and expectations. This article presents findings from a survey conducted on a sample of 1030 students in Denmark on whom ECSI methodology is applied.

Our research might help universities to gain a better understanding of students, their perceptions about various aspects of university and their expectations. It might assist universities to find ways to achieve high level of student satisfaction that leads to their loyalty and eventually to retention, through learning their behaviour, which will improve university’s performance.

## Theoretical background

### European customer satisfaction index model

**Fig 1:**
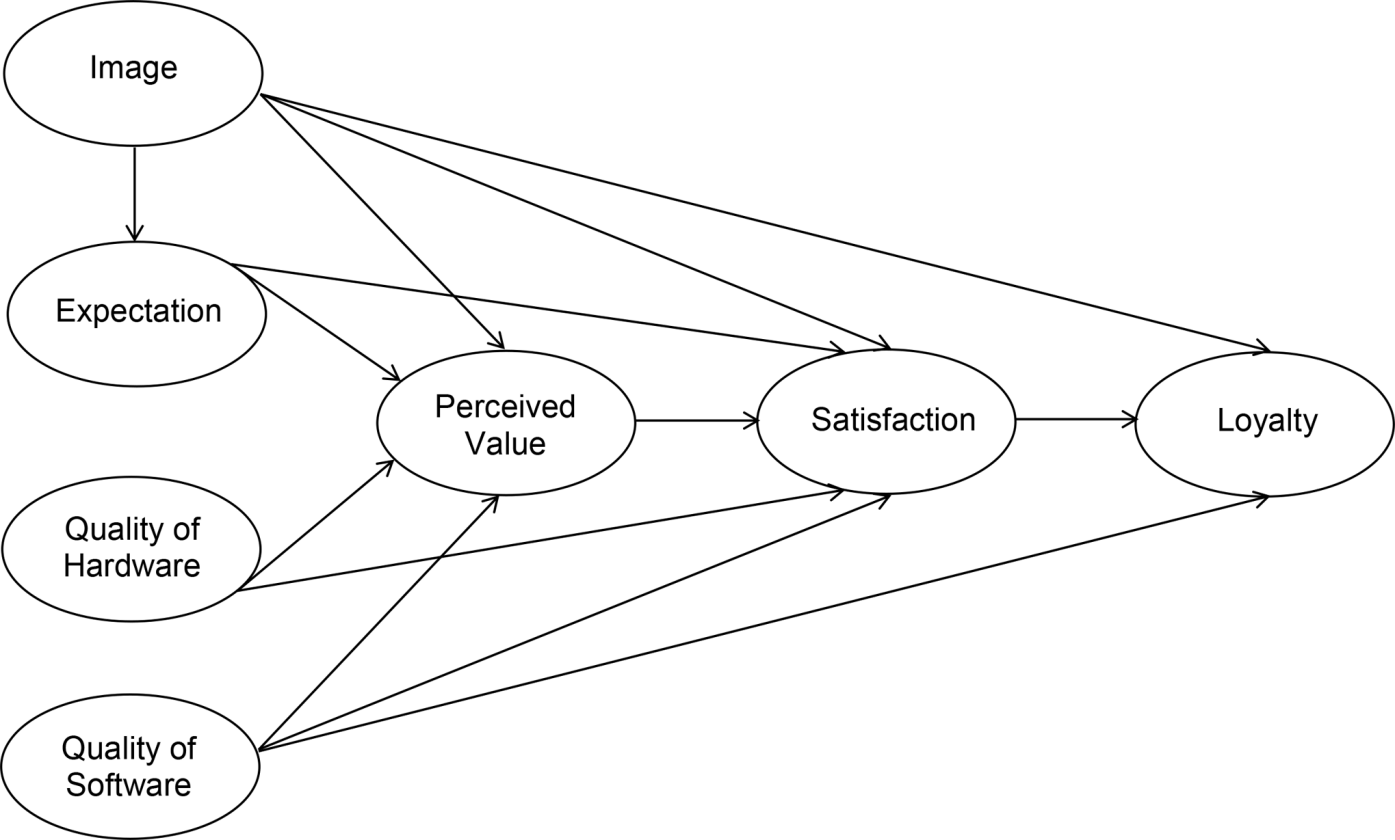
The research model.

Customer satisfaction indices (CSI) were built upon a cumulative view of satisfaction. CSI is a structural model based on the assumptions that customer satisfaction is caused by a number of factors such as perceived quality, perceived value, expectations of customers, and image of a firm. These factors are the antecedents of overall customer satisfaction. The model also estimates the results when a customer is satisfied or not. These results of customer satisfaction are consequences factors such as complaints or loyalty of customer [15]. Among four main wide measurement models of Swedish Customer Satisfaction Barometer (SCSB), American Customer Satisfaction Index (ACSI), Norwegian Customer Satisfaction Model (NCSI), and European Customer Satisfaction Index (ECSI) that are perceived as pioneering, ECSI is chosen for the aim of this research. These four nationwide measurement models lay ground for further development of satisfaction models that have been developed in different countries and continents. ECSI has been developed by results of a research carried out in 1998, which defines the satisfaction concept empirically and helps to understand the relationships between antecedents of satisfaction and their consequences such as loyalty and retention. ECSI is the most popular model as it is the most developed one among others [16]. The use of ECSI methodology to measure students’ satisfaction towards the higher education institutions attended was first presented in 1999 [17, 18] where the ECSI model together with an associated measurement instrument of students’ perceived quality, satisfaction and loyalty was developed and applied [18]. As other examples of applying satisfaction measurement models in the higher education context, Anne M. D.’s study in 2001 in which the comparative research method was adopted to analyse various student satisfaction indices in Babson College and five other colleges with different types, and Bruno Chiandotto’s study in 2004 in which ECSI was applied to implement quality evaluation to the college education process through investigating college students’ satisfaction to those college students in one year after graduation can be mentioned [19]. ECSI also has been used in 2005 by Østergaard and Kristensen in order to estimate drivers’ effects of student satisfaction and loyalty at different levels of higher education at Aarhus School of Business in Denmark [4]. Further, China scholars investigated the student satisfaction evaluation, and Liuwu and Yangxue in 2006 added the quality factor in the student satisfaction index model [19]. Robert M. Brown in 2006 conducted a research in Australian universities based upon ECSI to estimate factors driving student satisfaction and loyalty especially the institutional image of Australian universities [20]. Wanmin in 2007 also considered the analysis of student satisfaction from the higher education service process. Surveying tourism graduates in Portugal in 2012 is another example of applying and developing ECSI in the higher education context by measuring the effects of employability [17]. ESCI has been renamed to EPSI, which stands for European Performance Satisfaction Index. Based on recommendations from a feasibility study and the work provided by the ECSI technical committee, the EPSI rating framework for measuring customer satisfaction and customer loyalty was designed [21]. This allows opening in for other performance measures like employee satisfaction and society trust. The EPSI Rating is run under the umbrella of a European non-profit organisation, associated throughout its history and by its approach with the following leading European quality organisations EFQM (European Foundation for Quality Management), EOQ (European Organisation for Quality) and IFCF (International Foundation for Customer Focus) [21]. Our research model is based on the previously discussed versions of EPSI model. The introduced modification has an additional path between image and expectation. The modified research model with the core of the customer satisfaction model is illustrated in Fig 1.

### Image

Image is known as a perceptual phenomenon that is formed by rational and emotional interpretation and that has cognitive components, the beliefs, and affective components, the feelings [22]. Consequently, the overall image will be formed subjectively through a system consisting of these two components [23]. Series of hypotheses have been verified in empirical work of Palacio in university context; “*i*) the cognitive component of university image influences the affective component of that image, *ii*) the overall university image is formed through its cognitive and affective components, which is more influenced by the affective components than by the cognitive ones, *iii*) defined dimensions of the cognitive component of image influence the student satisfaction, *iv*) the affective component has more influence on the student satisfaction than the cognitive component, and v) the overall image also influences student satisfaction” [24].

To accomplish results of image effects in the university context, investing on plans and strategies related to the attributes that have the most influence on the affective and overall image of the university and satisfaction is required. Chosen plans and strategies should also consider the factors related to ‘the orientation towards, and the preparation of, students’, the ‘reputation’ and ‘ease of entrance to the university’. On the other hand cognitive attributes that almost do not have any influence on the affective and overall image should be avoided, which are elements that deal with ‘overcrowding’ and the ‘maturity/youth’ of the university. Thus, university’s brand image will have thorough foundations that are significant to one of its main publics, the students, whose satisfaction relies upon the perceived image. It will have the capacity to extend a corporate image in which the advantages related to that public are provided in a distinguished manner. [24].

In this study universities’ reputation, reliability and trustworthiness, being engaged in commercial and social conditions, being a place of new thinking, establishing contracts to Danish business and industry, being an internationally open university, efforts to meet students demands and easily adapting to the surrounding society have been considered to measure university’s image.

### Expectation

Students usually have profoundly different perception of the same experience when evaluating the courses and universities. Expectations that students have about the course or university prior to starting is one of the drivers that makes differences in students’ satisfaction. Therefore, as satisfaction is the outcome of researchers’ efforts, considering the role of expectation in students’ satisfaction and how it affects satisfaction are unavoidable [25].

In order to confirm the relationship between student satisfaction and student expectations, a paradigm known as expectancy/disconfirmation is presented, which has been studied and examined by many researchers and served as the basis for the vast majority of satisfaction studies that have been completed in the marketing and consumer behaviour fields [26–32]. The expectancy/disconfirmation paradigm includes four constructs: expectations, performance, disconfirmation, and satisfaction. Disconfirmation occurs when there are differences between prior expectations and actual performance or in other words perceived reality. In this situation three possible outcomes may happen; *i*) zero disconfirmation happens when perceived reality is as expected, *ii*) positive disconfirmation results when perceived reality is greater than expectations, which causes in satisfaction, and *iii*) negative disconfirmation happens when perceived reality is below expectations, which results in dissatisfaction [25].

Effects of expectations on satisfaction depend on when assessment happens. However, recall of expectation can be shaped by levels of student satisfaction. The key finding in Appleton-Knapp and Krentler’s study of expectation on student satisfaction (2006) is that students’ recall for what they expect from a course can be biased, which is linked to the timing of students’ assessments. When the hindsight bias repeats, then teachers should consider arranging those timing about assessments and appraisals to guarantee that they are eliciting precise response. For instance, feedback evaluations should be carried out immediately after the end of each course or midterm instead of one global evaluation at the end of the term. All in all, by helping students to establish proper expectations, student satisfaction could be increased, which is critical in order to improve learning and the accomplishment for guidelines objectives [25].

To measure expectation construct, what students expect from the structure of the programmes and the range of courses offered, the practical facilities, the lecturers’ teaching ability and contribution in general, and the service of administrative staff have been considered in this study.

### Quality of service

Due to the subjective nature of service quality [30], the service marketing literature focuses on quality in terms of perceived service quality [33]. The comparison of customer service expectations with their perceptions of actual performance results in perceived service quality [34], and is seen as a global judgement, or attitude, relating to the superiority of the service [35]. Later, Asthiyaman (1997) has extended the notion to overall evaluation of the goodness or badness of a product or service as perceived service quality. In addition, service quality is also defined as ‘the difference between what a student expects to receive and his/her perceptions of actual delivery’ [36]. Students’ perceived service quality is considered as an antecedent to student satisfaction [37, 38]. One important issue in higher education context is fluctuation of service quality perceptions over the time. Students’ experiences are varied and continuous by the time passes, which emphasizes the relevance of the context when measuring perceived service quality. Therefore, the perception of service quality not only depends on the service provider, but also on the performance of the consumer.

The key quality attributes as identified by Martensen et al. (1999) are split into quality of ‘human ware’ or ‘software’ and quality of ‘hardware’ in the higher education context. In the context of higher education, ‘human ware’ includes human elements as teaching, academic standard, pedagogical methods, and personal contact with teaching staff and administrative staff. The ‘hardware’ includes non-human elements as provided study programmes and courses and support functions (classrooms, library, computer facilities, equipment, student office, etc.) [18].

In order to measure quality of service from students’ perception at their university, we divided questions into two parts; *i*) questions with indicators that measure quality of human-ware were quality of service rendered by the administrative staff, quality of the lecturers’ teaching and contribution in general, and whether they met students’ demands on quality, and *ii*) questions with indicators that are designated to quality of hardware were the quality of structure and selection of courses offered in students’ programme, the quality of facilities and framework, and whether they met students’ demands on quality.

### Perceived value

From students’ perspective, perceived value is the overall assessment of utilizing the service according to their perception of what is received instead of what is given. The possibility of reaching objectives that students pursue during studying at the university also reflects the value of education [39].

There are areas of agreement that perceived value is based upon a comparison between benefits and sacrifices [40–44], is a preferential judgment [39], varies over time and location [39, 45, 46], contains a perceptual dimension [40, 46, 47], includes the objective or target the consumer seeks to attain through consumption [47, 48], depends on individual characteristics [49, 50] and displays a comparative character [39, 46, 51].

Satisfaction discloses the level of students’ fulfilment with the university’s services while value specifies which direction the university should take to achieve this satisfaction. By that it means where resources should be allocated when planning a service and how to design the interlinking of services in order to accomplish the desired results [52].

The evaluation of benefits that students gained, considering the time and personal resources spent on their studies, and the possibility of achieving the ability to manage their future career are the elements we used in this study to measure their perceived value of their university.

### Satisfaction

Satisfaction happens in higher education when perceived performance meets or exceeds the student’s expectations [8], which is being shaped continually by repeated experiences in campus life [8]. Pleasure is expected by students with the everyday process of learning [53, 54]. In other words, students’ demands and expectations are usually based mainly on their short-term goals, which is due to the incomplete understanding of what constitutes a quality education. Meeting such expectations could turn out to be detrimental to both students and the institution for the long term [54].

Motivation of entering the university for most students is to advance their education in order to improve their future career, which is a decision that also has impacts on students’ choice of university and study programme [55, 56]. Satisfaction conceptualisation in higher education varies depending on viewpoints that have been used in different studies [57–59]. For some researchers, satisfaction conceptualisation is viewed as a ‘process’ relating to its main causes [6, 60–64], or as a ‘result’ relating to its nature [53, 65]. However, student satisfaction as a process is more often used in studies. In order to avoid consequences of concerning about processes more than outcomes, universities can help students to clarify their long-term goals and expectations [64].

### Loyalty

Fernandes, Ross and Meraj (2012) confirmed the work done by Kotler and Fox (1995) and Helgesen and Nesset (2007) by proving that loyalty is positively related to student satisfaction, which increases performance and profitability in the long run [66, 67]. Therefore, having satisfied students will lead to loyalty behaviour [68, 69]. The positive relationship between satisfaction and loyalty reveals that satisfied students with their overall programme experience and with the quality of university facilities and services are more probable to be loyal to the university. This loyalty will lead to positive word of mouth, endorsing the university in the wider community and more desire in enrolment of graduates. Consequently, this positive word of mouth provides credibility to the university [70]. Webb and Jagun (1997) defined the concept of loyalty in the higher education context as student’s willingness to recommend the university/institution to others, the wish to tell positive things about the university/institution and their returning willingness to the university/institution in order to continue their studies [71]. From Athiyaman’ opinion, loyalty is the combination between students’ willingness to talk positively about the institution and to provide information to new candidates [72].

Students making use of the offers of further and supplementary programmes, conferences, etc. at their university after graduation, recommending their study programme and their university to others, and whether they would choose their programme and their university again with a free choice were the factors chosen in this study to measure students’ loyalty.

### Data and methodology

In this study, quantitative data have been collected by questionnaires in April 2014 from Danish universities. The questionnaire, which is provided in the S1 Text, was developed based on the drivers of student satisfaction and loyalty at different levels of higher education (HE) survey questions, which were used by Østergaard and Kristensen in 2005 at Aarhus School of Business [4]. Furthermore, 13 satisfaction questions from the Noel-Levitz questionnaire developed by the Office of Institutional Research were added to the questionnaire used in this research. The survey contained seven demographic questions. For the remaining questions, a 5-point Likert scale was used. Either 5-, 7- or 10-point scales are all comparable for analytical tools such as confirmatory factor analysis or structural equation models. Dawes suggests that none of the three formats is less desirable from the viewpoint of obtaining data that will be used for regression analysis. Kurtosis and skewness were likewise all very similar for each format [73]. In order to be compatible with the scales used in Østergaard and Kristensen’s study (2006) same scaling is used.

A probability sampling method was used to collect data in this research. The questionnaire was distributed as hard copies as well as online via SurveyXact. 50 hard copies of the questionnaire were distributed at the library of Technical University of Denmark (DTU) during one week. All the students from different faculties going to the library in that period had the chance to fill out the questionnaire. Meanwhile the online questionnaire was distributed through Facebook groups of various Danish universities (Aalborg University with 4800 members, Aarhus University with 6500 members, and MSc IBE/IM AAU 2012/14 with 85 members), of which 32 members responded. As there are a lot of spams on Facebook, it is assumed that people do not have desire to pay attention to unfamiliar posts and links. Questionnaire was also sent to Copenhagen University’s students' Email addresses within a month from the beginning of April 2014 and 948 responses were collected. Students of all the faculties are registered in Copenhagen University’s internal database, KUnet, and had the possibility to participate in the survey. After data screening and excluding the respondents who partially completed the questionnaire, 1,030 acceptable responses were collected from Danish universities. Altogether 1852 non-unique people opened the online questionnaire or filled in the paper questionnaire. Of these 1852, 666 only clicked on the questionnaire link but did not submit even answers for questions on the first page. Unlike e.g. Qualtrics, SurveyXact does not track IP addresses, so even if students clicked on the questionnaire link from home, i.e. from a unique IP address, it is not possible to identify how many of these 666 returned later and filled in the whole questionnaire and how many simply decided not to fill it in and did not return again. From 156 incomplete questionnaires, 58% of respondents dropped after the first page out of 4-page questionnaire. Majority of the excluded questionnaires contained only the answers for the first five questions. The remainder of excluded questionnaires were not finalized. None of the fully filled-in questionnaires were excluded. The questions about satisfaction and loyalty were placed at the end of the questionnaire. SurveyXact and paper do not offer a possibility to randomize order of the questions. Therefore, it was not possible to calculate even a partial model with not fully filled in responses. Since Likert scales were used, it did not make sense to search for outliers (identified by their distance from mean being a certain multiple of a standard deviation). Overall, it may be stated that answers were of good quality as answers correlated enough to fulfil criteria for being used as latent variables.

The distribution of respondents by gender, nationality and degree is provided in Table 1.

**Table 1:** General information about the respondents.

*General information about the respondents*		Associate Degree	Bachelor Degree	Master Degree	Doctorate or Professional Degree	Other	Total
Female	Danish	2.91%	29.51%	14.27%	0.58%	1.26%	48.53%
	International	0.39%	3.79%	8.35%	0.97%	0.29%	13.79%
Male	Danish	1.75%	18.25%	7.57%	0.29%	0.68%	28.54%
	International	0.39%	1.75%	5.83%	1.07%	0.10%	9.14%
Total		5.44%	53.30%	36.02%	2.91%	2.33%	100%

The ECSI model and the associated estimation procedure (Partial Least Squares (PLS)) allow calculation of indexes to measure student perception of generic exogenous latent variables (drivers) such as institution image, expectations, quality of human and non-human elements of teaching and learning, and endogenous variables such as perception of value, student satisfaction and loyalty [4].

Our developed version of the ECSI structural model of student satisfaction and loyalty, which is illustrated in Fig 1, is tested by PLS methodology using the second version of ‘SmartPLS’ software. Missing values were coded as 6; mean replacement was set (though not actually used) as the missing value algorithm for both PLS and bootstrapping. The PLS algorithm uses the path weighting scheme (the default setting for the weighting scheme) and the default setting ‘Mean 0, Var 1’ for the data metric. In bootstrapping setting, individual changes as sign changes has been chosen. In the sample size settings, the value 1030 was entered, as it is the number of cases in the dataset. Bootstrapping is essentially a (pseudo-)random simulation. Meaning that the elements in these resamples vary slightly, therefore by repeating bootstrapping calculation result values will be slightly different [74].

PLS estimation procedure of structural equation models has three stages of measurement model evaluation, structural model evaluation and overall SEM evaluation. At the first stage, three different criterions required to evaluate measurement models are reliability, convergent validity, and discriminant validity. Factor loadings’ coefficients for reliability and construct validity, Cronbach’s Alpha coefficients and composite reliability for internal consistency of the research model are reviewed carefully. Our measurement models are reflective as indicators of each latent variable are highly correlated. After inspecting factor loadings’ coefficients of each latent variable, only students’ expectations of ‘the lecturers teaching ability and contribution in general’ and ‘the service of administrative staff’- indicators’ loadings of expectation construct—in this research model were not reliable, therefore have been removed from the model. In general, items with loadings of less than 0.4, which is a threshold commonly used for factor analysis results, should be dropped [75]. Even when the researcher has a strong theoretical rationale for including such items in the model, items with extremely low loadings should be reviewed carefully, since they will add very little explanatory power to the model while attenuating and therefore biasing estimates of the parameters linking constructs [76].

In this study, several indicators designated to the satisfaction construct were dropped out to improve quality of the research model after validity and reliability tests on measurement models. The indicators remained in the research model were included in questions concerning the students’ expectations in the university in general and in different aspects and the closeness of their university to an ideal one, the extent of which their expectations were met in general and related to different aspects such as teaching staff caring about the students, being fair and unbiased, taking students differences into consideration, their availability, and being effective communicators, how knowledgeable, caring and helpful campus staff were and the sense of belonging.

Next, convergent validity that is based on the correlation between responses obtained by maximally different methods of measuring the same construct is evaluated by average variance extracted (AVE) [77]. Discriminant validity as the third criterion confirms the validity of the latent constructs and their respective indicators. The cross loadings include determinant values that indicate correlations between observed items and their latent variables, which have to be higher than their correlations with other latent variables/constructs. Evaluation and confirmation discriminant validity of measurement models are also tested through a matrix suggested by Fornell and Larcker (1981) [78]. Based on their suggestion, the square root of latent variables’ AVE has to be higher than inter construct correlations.

At the second stage, relationships between the research model’s constructs are evaluated at the structural level. In this regards, t-statistic values for significance testing of structural paths and R-square values for predictability power of endogenous constructs are compared to what has been provided [79, 80]. Stone-Geisser’s Q2 criterion, which measures predictive relevance of the model and the ability of the model to provide a prediction of the endogenous latent variable’s indicators, is also estimated [81, 82].

At the third stage, a global criterion for goodness of fit (GoF) proposed by Tenenhaus for PLS-SEM analysis is calculated. This index as a single measurement formula has been developed to consider both measurement and the structural models and evaluates overall prediction of the model at once [83].

## Results

As the first stage of testing the research model in ‘SmartPLS’, factor loadings’ coefficients were inspected. The PLS model with all statements had three loadings of less than 0.4. The variables with loadings of less than 0.4 had been excluded one by one as suggested by Hulland (1999) to improve the model. Therefore, ‘the lecturers’ teaching ability and contribution in general’ and ‘the service of administrative staff’ from students’ expectations perspective and students’ satisfaction of their expectation being met by ‘help and approach of library staff’ were dropped at this stage. Cronbach’s Alpha, composite reliability, AVE and Stone-Geisser’s (Q^2^) values for the improved constructs are provided in Table 2.

**Table 2:** Model validation values.

	AVE	Composite Reliability	Cronbach’s Alpha	Communality	Redundancy	1-SSE/SSO (Q^2^)
Expectation	0.728799	0.842735	0.634903	0.728799	0.020406	0.019734
Hardware	0.581904	0.847424	0.766960	0.581904		
Image	0.474491	0.876657	0.839601	0.474491		
Loyalty	0.612767	0.885613	0.836419	0.612767	0.365822	0.360887
Satisfaction	0.457295	0.901886	0.882972	0.457295	0.299985	0.287103
Software	0.558694	0.832646	0.749501	0.558693		
Value	0.763080	0.865557	0.691147	0.763080	0.278524	0.274346

Cronbach’s Alphas for five out of seven constructs are greater than 0.7 the threshold suggested by Nunnally (1978) [76]. Cronbach’s Alpha for perceived value is 0.69, i.e. almost 0.7. Cronbach’s Alphas for expectation is 0.63, but since Cronbach Alpha is not a good measure of internal consistency when a scale consists of only a few items [84], the value of 0.63 should be rather compared to 0.50, the threshold suggested by Coulston (2008) for scales consisting of three or four items [85].

All AVEs are greater than 0.4, the threshold suggested by Magner, Welker, & Campbell (1996) and all composite reliabilities are higher than 0.7, the threshold suggested by Nunnally (1978) [76, 77]. Next, based on the condition suggested by Fornell and Larcker (1981) [78], four variables were excluded from the satisfaction construct because the square root of satisfaction’s AVE was lower than correlation between loyalty and satisfaction in the matrix. In this regards, the four excluded statements are whether students’ satisfaction based on their expectations are met by ‘teaching staff providing timely feedback about student’s progress in a course’, ‘supervisor being concerned about student’s success as an individual’, ‘supervisor being knowledgeable about requirements in student’s major’, and ‘student’s supervisor being helpful and set goals to work toward’ had been excluded from the model consecutive.

**Fig 2:**
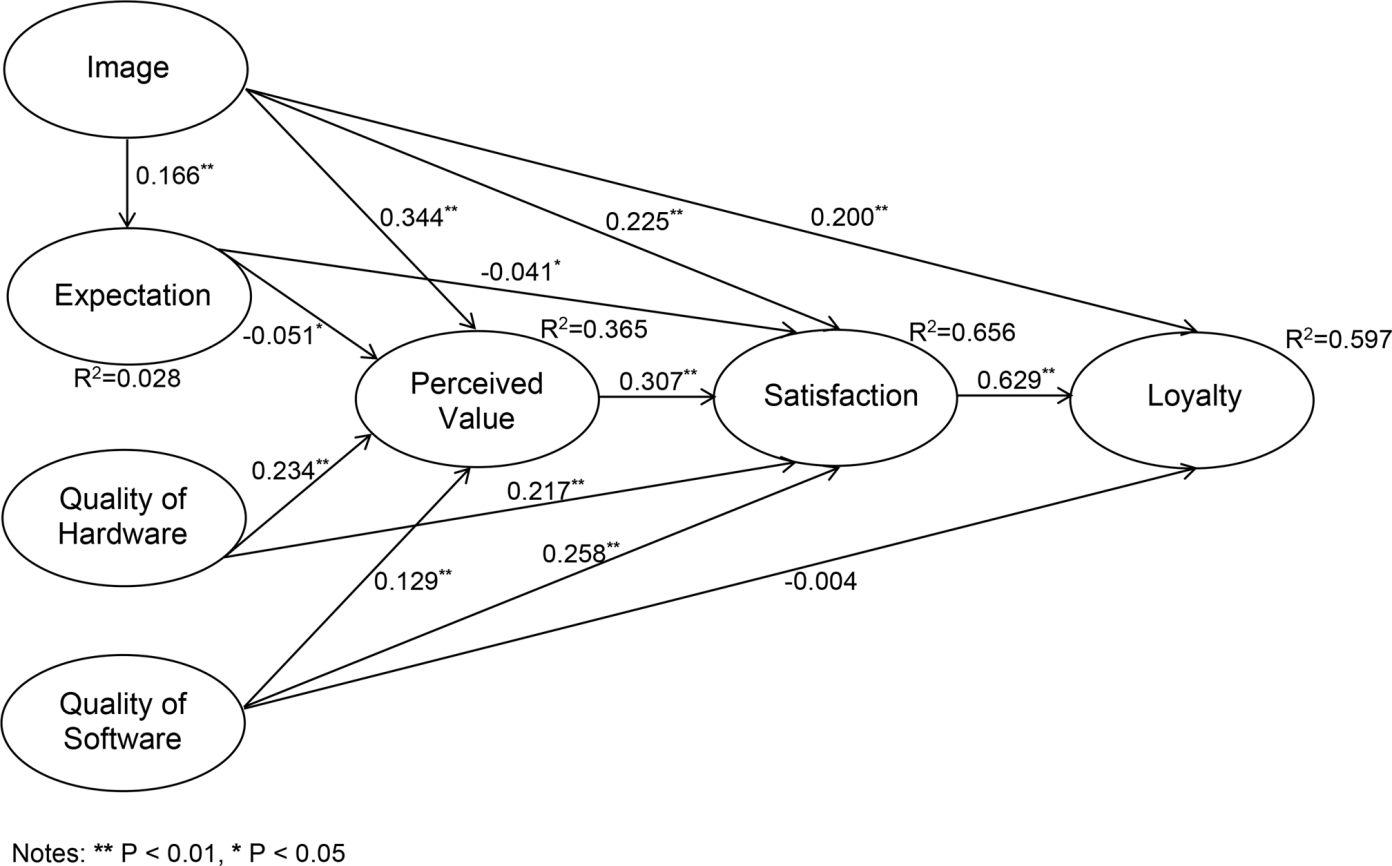
Significance of path coefficients of the model.

The PLS model is provided in Fig 2, which contains R^2^ and path coefficients. Path coefficients in SmartPLS correspond to standardized regression coefficients; they show which latent variables have a greater effect on the dependent variables. The model’s Goodness of fit index is calculated as 0.496.

All direct effect values (F^2^) of the research model are provided in Table 3. In this table, exogenous constructs are placed in columns and endogenous constructs are in rows. Indirect effects can be found in the S1 Table where indirect effects of ‘image on satisfaction through expectation’, ‘image on perceived value through expectation’ and ‘expectation on satisfaction through perceived value’ are valid at the confidence level of 90%. The rest of indirect effects in the S1 Table are accurate at the confidence level of 95%.

**Table 3:** Effect sizes of the research model.

	Image	Expectation	Hardware	Software	Value	Satisfaction
Value	0.105512	0.003149	0.045669	0.015748	NA	NA
Satisfaction	0.075581	0.005814	0.069767	0.101744	0.183139	NA
Loyalty	0.059553	NA	NA	- 0.007444	NA	0.421836

As shown in Table 3, the effect size of satisfaction on loyalty is large (i.e. over 0.35) and the effect size of perceived value on satisfaction is medium (i.e. over 0.15). The remaining effect sizes are weak. The effect size of ‘quality of software’ on loyalty is approximately zero. Therefore, quality of software does not have an impact on loyalty in Danish universities at the structural level of the model.

## Discussion

Dropping out of the study programme, which is one of the consequences of students’ dissatisfaction, affects both costumers and investors of higher education market place. In Denmark, in which this study took place, this will imply a negative impact on both the individual and the society, which is against the main goals of the ministry of higher education and science [14].

In this study, the role of different drivers of student satisfaction and loyalty was investigated using the ECSI methodology in the higher education marketplace. As students and employers think globally, European universities need to do so as well. They have to consider that technology changes the possibilities of learning, both in time and space, creating new market conditions for universities, which make student satisfaction and loyalty more essential to study.

Results of our research provide a more precise perspective on students by covering more satisfaction statements compared to a previous study [4, 86]. Furthermore, the questionnaires were conducted while students were experiencing and evaluating the service: that is in the act of studying. We have investigated the influence of university’s image on student’s expectations, which adds a new dimension to the model, while our results further confirm several findings of previous studies of student satisfaction [70, 87–90].

Elliott (2002) showed that ‘providing quality education’ and ‘feeling of belonging’ are key determinants of student satisfaction. In addition, he also stated that ‘students need to experience intellectual growth … and interact with faculty who are fair/unbiased and are able to provide quality instruction … Students want to expand their general knowledge and understanding in the areas they are majoring in’ [88]. These elements had significant effects on satisfaction in our research as well. However, ‘experience based satisfaction’, ‘expectations fulfilment’ and ‘the difference from ideal university’ were the most determining factors of satisfaction in our model. Examining the impact of image and expectations on satisfaction in the process of student satisfaction formation in higher education, our model shows a negative impact of expectation on student satisfaction meaning that high expectations reduce the level of student satisfaction, which is in line with Alves’s and Raposo’s (2007) findings. Based on their research, university’s image influences the formation process of student expectation, satisfaction and loyalty, which requires having knowledge of university’s image and measuring it. Students’ expectations should also be managed carefully in a way not to raise too much expectations that results in lack of satisfaction [89].

Furthermore, Fernandes, Ross and Meraj in 2012 confirmed Schneider and Bowen (1995) and Bigne et al. (2003) work in regards to the importance of the quality of teaching [70, 91, 92]. They proved that a significant level of satisfaction with overall programme quality could be attributed to the role of teaching staff in order to keep students satisfied with their programmes [70]. Also in our research, ‘quality of the lecturers’ teaching and contribution in general’ was significant, actually the strongest one. ‘Quality of software’ achieved the second strongest effects on satisfaction.

Other significant antecedents of satisfaction have been proven to be academic support, advice, organisation and management of students’ programme. Gardiner in 1998 and later Davis in 2001 have demonstrated that academic support and advising are powerful tools for universities to help students to be successful and to improve satisfaction with their experience [70]. It can be seen that a wise decision for universities is ‘to constantly improve the quality of educational programmes at all levels and to strive for high standards in course content and pedagogy’ [37]. Students will be satisfied when they receive a quality education in their field with an accompanying credential that is valued in the labour market [90]. Furthermore, one of the key components that affects students’ persistence is the student involvement in the college/university. Astin’s (1993) research and later Heverly’s (1999) study on new on-campus students confirmed that returning students between fall and spring semesters to their institution are more satisfied students having positive interactions with the faculty [63, 87].

Our study demonstrates that among constructs that are identified as antecedents of satisfaction, perceived value is the most important factor for students to be satisfied with their experience in Danish universities. The second most important construct appeared to be students’ perceived quality of software, of which 13.3% of this total effect is caused indirectly through perceived value. Furthermore, students’ perception on value received was mostly affected by university’s image. In this research, the possible relationship between university’s image and students’ expectation (that is assumed to have indirect effects on the endogenous construct of the model) has also been investigated. The results show that there is a significant relationship between image and expectations. There are also significant indirect effects of university’s image on students’ satisfaction and perceived value *via* students’ expectations in Danish universities. Our results show that 2.4% of the total effect of image on perceived value is explained by the indirect effect via students’ expectations. A VAF value of 2.93% indicates that a negligible total effect of image on satisfaction is explained by the indirect effect *via* students’ expectations. Our results also demonstrate that approximately half of the total effects of image on loyalty come indirectly from students’ satisfaction with Danish universities. Another highlight of the results is the magnitude of the indirect effect of image *via* perceived value on students’ satisfaction in Danish universities that is illustrated by the VAF value of 31.94%. Looking more into details, the most effective reason for students’ satisfaction is the way that students feel about their university or in other words their sense of belonging to their university. In Denmark, students’ evaluation of benefits they receive from studying at their university based on the time and personal resources they spent appeared to be the most important factor in perceived value which considering it will have positive influence on student satisfaction. Consequently, these results offer valuable insights for universities’ managers. A few examples for Danish universities in order to make the learning experience more of a partnership with students are designing student-oriented curricula, teaching guarantees [93], increased student input in policy formulation that causes higher quality instruction, greater accessibility of the faculty [94], and more efficient processes at every level of the university community [90, 95]. Involving students in the learning experience will consequently increase loyalty, which will lead to retention at the universities of higher education. Result of our research also confirms the fact that the most effective factor that influences positively on students’ loyalty is students’ satisfaction meaning the more satisfied students will be the loyal ones. However, perceived ‘quality of software’ as the second most important driver of students’ satisfaction, which includes lecturers’ teaching ability and contribution, service rendered by the administrative staff, and whether these factors meet students’ demands on quality did not have significant direct effect on students’ loyalty.

Eventually, findings of this research may inspire managers of higher education institutions and universities in order to maintain and improve students’ experiences during their time at the university. Dedicating resources to improve students’ desires will result in university’s development in the competitive market place of higher education. Therefore, loyalty of students will also increase.

## Conclusion

Based on the EPSI model, university’s image, students’ expectation, perceived value, perceived quality of software and perceived quality of hardware are assumed to have direct and indirect impacts on satisfaction and loyalty. Likewise, the effects of these variables on student loyalty are estimated. The theoretical basis of this study considers the fact that same as other organisations and companies that recently adopted customer-oriented strategies, universities as the service providers in the higher education marketplace should also utilize a more consumer-oriented strategy to deliver their services and satisfying the wants and needs of their students more effectively.

This research confirms the validity of the EPSI-SEM application in the context of higher education that shows the implementation of more detailed analyses are required in the process of students’ enrolment. The results of the structural equation modelling procedure evidently demonstrate that the adapted EPSI methodology can reliably and consistently measure student satisfaction and loyalty in Denmark’s universities of higher education. For individual universities or institutions of higher education this model facilitates tracking performance over time, benchmarking, and diagnosing the effects of various quality initiatives, which require extension of the basic model [18]. In this work, effects of university’s image, students’ expectations, perceived quality of software, perceived quality of hardware and perceived value on satisfaction have been tested. University’s image, perceived quality of software and satisfaction that based on theoretical background of EPSI model has effects on loyalty have also been assessed.

Educational researchers and universities’ managers should classify influential factors of student satisfaction and loyalty based on their importance in the current environment, and allocate resources to improve university’s position in the competitive marketplace of higher education in order to attract more funds and students. Furthermore, identifying students’ demands and fulfilling them are essential to make students satisfied and loyal and to become leaders in the higher education market. In addition to findings of this study, further research is required to investigate effects of demographic and personality characteristics of students in order to make informed decisions and remain successful in the competitive marketplace of higher education.

Countries like Denmark, Sweden, Norway and Finland share cultural similarities, as they were one Empire. Additionally, public universities are tuition free in Scandinavian countries for EU/EEA students; we therefore believe that our model is applicable in other Scandinavian countries, especially Norway and Sweden.

## Supporting information

S1 Table Indirect effects. (DOCX)

S1 Text Questionnaire. (DOCX)
