# Supplementary material for: Student satisfaction and loyalty in Denmark: Application of EPSI methodology
Source: PLoS One. 2017 Dec 14;12(12):e0189576. doi: 10.1371/journal.pone.0189576 (PMC5730189; doi:10.1371/journal.pone.0189576)
Supplement: S1 Table — (DOCX) [file pone.0189576.s001.docx]

**Indirect Effects**

| **Image, Expectation, satisfaction** | | | |  | **Image, Expectation, value** | | | |
| --- | --- | --- | --- | --- | --- | --- | --- | --- |
| **Variable** | **Value** | **Z-value** | **VAF** |  | **Variable** | **Value** | **Z-value** | **VAF** |
| a | 0.166 | 1.813666^*^ | 0.029361 |  | a | 0.166 | 1.73893^*^ | 0.024019 |
| b | 0.041 |  |  |  | b | 0.051 |  |  |
| S_a_ | 0.032554 |  |  |  | S_a_ | 0.032554 |  |  |
| S_b_ | 0.020733 |  |  |  | S_b_ | 0.027055 |  |  |
| c | 0.225 |  |  |  | c | 0.344 |  |  |
|  | | | |  |  |  |  |  |
| **Image, value, satisfaction** | | | |  | **Image, satisfaction, loyalty** | | | |
| **Variable** | **Value** | **Z-value** | **VAF** |  | **Variable** | **Value** | **Z-value** | **VAF** |
| a | 0.344 | 7.334618^**^ | 0.319436 |  | a | 0.225 | 6.682994^**^ | 0.414391 |
| b | 0.307 |  |  |  | b | 0.629 |  |  |
| S_a_ | 0.038033 |  |  |  | S_a_ | 0.032077 |  |  |
| S_b_ | 0.024344 |  |  |  | S_b_ | 0.028301 |  |  |
| c | 0.225 |  |  |  | c | 0.2 |  |  |
|  |  |  |  |  |  |  |  |  |
| **Expectation, Value, satisfaction** | | | |  | **Hardware, Value, satisfaction** | | | |
| **Variable** | **Value** | **Z-value** | **VAF** |  | **Variable** | **Value** | **Z-value** | **VAF** |
| a | 0.051 | 1.858629^*^ | 0.276347 |  | a | 0.234 | 5.616269^**^ | 0.248714 |
| b | 0.307 |  |  |  | b | 0.307 |  |  |
| S_a_ | 0.027055 |  |  |  | S_a_ | 0.037188 |  |  |
| S_b_ | 0.024344 |  |  |  | S_b_ | 0.024344 |  |  |
| c | 0.041 |  |  |  | c | 0.217 |  |  |
|  |  |  |  |  |  |  |  |  |
| **Software, Value, satisfaction** | | | |  |  |  |  |  |
| **Variable** | **Value** | **Z-value** | **VAF** |  |  |  |  |  |
| a | 0.129 | 3.439024^**^ | 0.133073 |  |  |  |  |  |
| b | 0.307 |  |  |  |  |  |  |  |
| S_a_ | 0.035976 |  |  |  |  |  |  |  |
| S_b_ | 0.024344 |  |  |  |  |  |  |  |
| c | 0.258 |  |  |  |  |  |  |  |

Note: ** p<0.05, * p<0.1
