# Supplementary material for: Student satisfaction and loyalty in Denmark: Application of EPSI methodology
Source: PLoS One. 2017 Dec 14;12(12):e0189576. doi: 10.1371/journal.pone.0189576 (PMC5730189; doi:10.1371/journal.pone.0189576)
Supplement: S1 Text — (DOCX) [file pone.0189576.s002.docx]

### Questionnaire

| ***Dear participant,***  I am a student of Aalborg University and willing to do a research on student satisfaction in Danish universities. I appreciate your dedicated time very much, which will help me to figure out how to make it a better place to study for all of us. Your answers will be treated as confidential and will not be handed over to a third party. The results of the questionnaire will only be presented collectively.  **How to fill out the questionnaire:**  The questionnaire contains 20 questions, please answer the following questions by crossing the relevant square () and/or writing down the answer in the provided space.  **Thank you in advance for your participation, your opinion is very important for me!**  Yours Sincerely,  Tina Shahsavar   |
| --- |

For further inquiries please do not hesitate to contact me:

Email: [tshahs12@student.aau.dk](mailto:tshahs12@student.aau.dk)

Tel: 71727477

| 1. Gender:  \| Female \| Male \| \| --- \| --- \| | 1. Age:  \| 18 and under \| 19 to 24 \| 25 to 34 \| 35 to 44 \| 45 and over \| \| --- \| --- \| --- \| --- \| --- \| |
| --- | --- | --- | --- | --- | --- | --- | --- | --- |
| 1. Nationality:   ____________________ | 1. Current Educational Status:  \| Associate degree \| Bachelor's degree \| Master's degree \| Doctorate or professional degree \| Other \| \| --- \| --- \| --- \| --- \| --- \| |
| 1. Where do you study?   ____________________ | 1. What is your study programme?   ____________________ |

1. **Employment (Multiple Choice):**

| Full-time Off Campus | Part-time Off Campus | Full-time On Campus | Part-time On Campus | Not Employed |
| --- | --- | --- | --- | --- |

1. To what extent do you agree with the following statements about your university?

|  | Strongly disagree | Disagree | Neutral | Agree | Strongly agree |
| --- | --- | --- | --- | --- | --- |
| The University's reputation is good | (1) | (2) | (3) | (4) | (5) |
| It is a place of new thinking | (1) | (2) | (3) | (4) | (5) |
| It is a reliable and trustworthy university | (1) | (2) | (3) | (4) | (5) |
| It is engaged in commercial and social conditions | (1) | (2) | (3) | (4) | (5) |
| It has established contracts to Danish business and industry | (1) | (2) | (3) | (4) | (5) |
| It is an internationally open university | (1) | (2) | (3) | (4) | (5) |
| It makes a great effort to meet students demands | (1) | (2) | (3) | (4) | (5) |
| It adapts easily to the surrounding society | (1) | (2) | (3) | (4) | (5) |

1. Considering your overall expectations, how important do you see the following factors in a university?

|  | Not important | Slightly important | Moderately important | Very important | Extremely important |
| --- | --- | --- | --- | --- | --- |
| The lecturers’ teaching ability and contribution in general | (1) | (2) | (3) | (4) | (5) |
| The service of administrative staff | (1) | (2) | (3) | (4) | (5) |
| The structure of the programmes and the range of courses offered | (1) | (2) | (3) | (4) | (5) |
| The practical facilities | (1) | (2) | (3) | (4) | (5) |

1. How do you assess the overall quality of the following issues at your university?

|  | Very poor | Poor | Fair | Good | Excellent |
| --- | --- | --- | --- | --- | --- |
| The structure and selection of courses offered in your programme | (1) | (2) | (3) | (4) | (5) |
| The facilities and framework | (1) | (2) | (3) | (4) | (5) |
| The structure and courses in your programme meeting your demands on quality | (1) | (2) | (3) | (4) | (5) |
| The facilities and framework meeting your demands on quality | (1) | (2) | (3) | (4) | (5) |
| The lecturers’ teaching and contribution in general | (1) | (2) | (3) | (4) | (5) |
| Service rendered by the administrative staff | (1) | (2) | (3) | (4) | (5) |
| The lecturers’ teaching ability and contribution in general meeting your demands on quality | (1) | (2) | (3) | (4) | (5) |
| The service rendered by the administrative staff meeting your demands on quality | (1) | (2) | (3) | (4) | (5) |

1. Considering the time and personal resources you spent on your studies, how do you evaluate your benefits from having studied at this university?

| Very poor | Poor | Fair | Good | Excellent |
| --- | --- | --- | --- | --- |

1. To what extent do you agree that studying at this university will give you the ability to manage your future job and career?

| Strongly disagree | Disagree | Neutral | Agree | Strongly agree |
| --- | --- | --- | --- | --- |

1. Based on your experience from studying at this university, how satisfied you are in general?

| Very dissatisfied | Somewhat dissatisfied | Neither dissatisfied nor satisfied | Somewhat satisfied | Very satisfied |
| --- | --- | --- | --- | --- |

1. How far/close is this university from your ideal university?

| Completely different | Very different | Moderately different | Slightly different | Not at all different |
| --- | --- | --- | --- | --- |

1. To what extent your expectations of the study programme have been fulfilled at this university?

| Not at all | To some extent | To an average extent | To a considerable extent | To a great extent |
| --- | --- | --- | --- | --- |

1. To what extent your expectations of this university have been fulfilled?

| Not at all | To some extent | To an average extent | To a considerable extent | To a great extent |
| --- | --- | --- | --- | --- |

1. **To what extent are you satisfied with your expectations being met relating to the following items at your university?**

|  | Very dissatisfied | Somewhat dissatisfied | Neutral | Somewhat satisfied | Very satisfied | No experience |
| --- | --- | --- | --- | --- | --- | --- |
| Teaching staff care about me as an individual | (1) | (2) | (3) | (4) | (5) | (6) |
| Teaching staff are fair and unbiased in their treatment of individual students | (1) | (2) | (3) | (4) | (5) | (6) |
| Teaching staff take into consideration student differences as they teach a course | (1) | (2) | (3) | (4) | (5) | (6) |
| Teaching staff provide timely feedback about student progress in a course | (1) | (2) | (3) | (4) | (5) | (6) |
| Teaching staff are usually available after class and during office hours | (1) | (2) | (3) | (4) | (5) | (6) |
| Most teaching staff on this campus are effective communicators | (1) | (2) | (3) | (4) | (5) | (6) |
| The campus staff are knowledgeable, caring and helpful | (1) | (2) | (3) | (4) | (5) | (6) |
| Library staff are helpful and approachable | (1) | (2) | (3) | (4) | (5) | (6) |
| My supervisor is concerned about my success as an individual | (1) | (2) | (3) | (4) | (5) | (6) |
| My supervisor is knowledgeable about requirements in my major | (1) | (2) | (3) | (4) | (5) | (6) |
| My supervisor helps me set goals to work toward | (1) | (2) | (3) | (4) | (5) | (6) |
| I feel a sense of belonging here | (1) | (2) | (3) | (4) | (5) | (6) |

1. How likely will you:

|  | Not at all likely | Slightly likely | Moderately likely | Very likely | Completely likely |
| --- | --- | --- | --- | --- | --- |
| Make use of the offers of further and supplementary programmes, conferences, etc. at this university after your graduation? | (1) | (2) | (3) | (4) | (5) |
| Recommend this university to others? | (1) | (2) | (3) | (4) | (5) |
| Recommend your study programme to others? | (1) | (2) | (3) | (4) | (5) |

1. If you had to study again, with a free choice of institution of education, how likely would you choose this programme?

| Not at all likely | Slightly likely | Moderately likely | Very likely | Completely likely |
| --- | --- | --- | --- | --- |

1. If you had to study again, with a free choice of institution of education, how likely would you choose this university?

| Not at all likely | Slightly likely | Moderately likely | Very likely | Completely likely |  |  |
| --- | --- | --- | --- | --- | --- | --- |
| Thank you very much for your participation! | | | | | | |
